# Outcome of conventional IVF and ICSI on sibling oocytes in moderate ligoasthenozoospermia 

**Published:** 2013

**Authors:** Bao-Guo Xie, Wei-Jie Zhu, Yuan-Hua Huang

**Affiliations:** 1 **Bao-Guo Xie, Department of Obstetrics and Gynecology, The first Affiliated Hospital of Jinan University, Guangzhou, China.**; 2 **Wei-Jie Zhu, Institute of Reproductive Immunology, Jinan University, Guangzhou, China.**; 3 **Yuan-Hua Huang, Department of Obstetrics and Gynecology, Center of Reproductive Medicine, The Affiliated Hospital of Hainan Medical College, Haikou, People’s Republic of China.**

**Keywords:** Oligoasthenozoospermia, Infertility, In vitro fertilization (IVF), Intracytoplasmic sperm injection (ICSI)

## Abstract

***Objective:*** To evaluate the outcomes of patients with moderate oligoasthenozoospermia treated with conventional in vitro fertilization (IVF) and intracytoplasmic sperm injection (ICSI).

***Methods:*** A total of 99 couples with moderate oligoasthenozoospermia undergoing their first IVF/ICSI cycle were included in the study. Sibling oocytes were randomized to be inseminated either by conventional IVF or ICSI. Fertilization rate, cleavage rate, embryo quality, implantation rate, and clinical pregnancy rate were examined.

***Results: ***There was no difference in the fertilization rate, cleavage rate, implantation rate, and pregnancy rate between conventional IVF and ICSI (P>0.05). The good quality embryo rate was significant difference between after IVF and after ICSI (P<0.05).

Conclusions: Couples with moderate oligoasthenozoospermia did not influence the major indices of IVF and the uncertainties concerning the safety of ICSI, couples with moderate oligoasthenozoospermia need not be subjected to ICSI.

## INTRODUCTION

Male factor infertility is responsible for half of the couples with infertility. Due to no uniform treatment criteria, decisions for couples with male infertility were often empirical and might lead to the failure after in vitro fertilization (IVF), or to the unnecessary use of intracytoplasmic sperm injection (ICSI).

Moderate oligoasthenozoospermia is usually defined as sperm count 5-9×10^6^ sperm/ml, which accompany <32% progressive motility. A significant effect of a single suboptimal semen parameter on the fertilization results after IVF and ICSI was reported for sperm progressive motility. Moreover, injection of motile spermatozoa into oocytes were the most important factor in determining good results with ICSI was also observed. These results indicated that conventional IVF or ICSI can gave the chance of having a child to infertile couples with male infertility. However, the choice of assisted reproduction (IVF or ICSI) for couples with moderate oligoasthenozoospermia has not been evaluated.

With the development of micromanipulation, ICSI has rapidly became an important part of choice for the treatment of male factor infertility. Although the ICSI is very successful, it potential risk is still undergoing evaluation. The potential risk of ICSI, including the possible asynchronized decondensation of sperm chromosomes, the lower survival, the lower implantation rates of frozen-thawed embryos originating from ICSI than of embryos obtained by IVF, and the malformations and chromosome abnormalities observed in the fetus. However, with the progress of semen preparation and embryo manipulation, the clinical pregnancy rate of conventional IVF has been increased steadily. Therefore, some authorities advocated that the use of ICSI only when previous fertilization failure with IVF has occurred, or the number and quality of available spermatozoa was not appropriate for IVF.

The aim of this study was to evaluate whether couples with moderate oligoasthenozoospermia should be treated with conventional IVF or ICSI. The results might help us to determine the optimal treatment (IVF or ICSI) used for couples with moderate oligoasthenozoospermia.

## METHODS

A total of 99 couples undergoing their first IVF/ICSI cycle were considered for this study between 2008 and 2012, and for whom at least 8 oocytes were retrieved. All couples had only moderate oligoasthenozoospermia, and none had a history of radiotherapy, chemotherapy, chronic illness, or medication. Male inclusion criteria were sperm count 5-9×10^6^ sperm/ml, which accompany progressive motility <32%. Patients were excluded in this study if the female with clinically abnormal female (ovulatory, tubal, endometrial, and cervical) factor, and female’s age was more than 35 years. Written informed consent was obtained from all couples. The women had to be aged between 26 and 35 years and a body mass index (BMI) of 23-29 Kg/m^2^. The project was approved by local ethics committee.

Fresh ejaculate samples were obtained by masturbation after 3-7 days’ abstention from sexual intercourse, and the semen samples were prepared after a minimum of 20 minutes liquefaction at room temperature. After liquefaction, sperm concentration and progressive motility were assessed according to the recommendations of the World Health Organization manual. All female partners underwent ovarian stimulation by a long desensitization protocol. Oocytes were retrieved by transvaginal ultrasound-guided follicle aspiration, 34-36 h after HCG injection. Only MII oocytes with extruded first polar body were micro-injected. Gamete manipulation for conventional IVF and ICSI was performed as the manual of IVF in our lab.

Normal fertilization was confirmed by the presence of 2 pronuclei (2 PN) and 2 polar bodies 16-20 h after IVF and ICSI. Cleavage and embryo quality were evaluated at days 2 and 3 after oocyte retrieval. Embryos were scored according to the number, size of the blastomeres and the amount of anucleate fragmentation: type 1, embryos had equal-sized blastomeres and anucleate fragmentation; type 2, embryos had blastomeres unequal in size and <20% fragmentation; type 3, embryos had 20%-50% fragmentation; and type 4, embryos had >50% fragmentation. Embryo transfer was performed 48-72 h after the oocyte retrieval. Two or three embryos were chosen for transfer to the female partners depending on their morphological quality. Details of the embryo transfer technique were described the previous study. Clinical pregnancy was defined as a positive HCG test in serum and the ultrasonographic demonstration of an intrauterine gestational sac 6-7 weeks after embryo transfer.

Statistically significant differences were determined using unpaired student’s *t*-test, Fisher’s exact test as appropriate. The outcomes were compared by using *x*^2^-test. A *P-*value <0.05 was considered statistically significant. All tests were performed by using the SPSS 15.0 (Chicago, IL, USA) statistical package.

## RESULTS

The clinical characteristics of couples are listed in Table-I: The age, BMI, and duration of infertility of patients who received IVF and ICSI were not statistically significant (P>0.05). No significant differences were also found between IVF and ICSI in other parameters, including serum day 3 FSH and E_2_, gonadotrophin does, total 2PN zygotes, number of oocytes and mature oocyte number (P>0.05). Moreover, the sperm parameters were also no significant difference between IVF and ICSI (Table-II).

The fertilization rate was low after IVF (70.1%) compared with after ICSI (71.9%), but this difference was not statistical significance (P>0.05). The good quality embryo rate was significantly better after ICSI than after IVF: 32.9% vs. 24.0%. However, the cleavage rate, implantation rate and pregnancy rate were lower after ICSI than after IVF: 97.4% vs. 94.75, 28.4% vs. 36.8%, 41.1% vs. 57.7%, respectively. These data are given in Table-III. Although no significant differences were found with regard to cleavage rate, implantation rate, and pregnancy rate between after ICSI and after IVF (P>0.05), the good quality embryo rate was significantly different after ICSI and after IVF (P<0.05).

**Table-I:** Characteristics of the treatment cycles

	***C*** ***onventional IVF***	***ICSI***	***P***
**No. of cycles**	**46**	**53**	
**Mean female age (years)**	**31.2** **±2.** **7**	**3** **1** **.** **1** **±** **2** **.** **0**	**NS**
**Mean male age (years)**	**33.** **5±** **2.8**	**33.6** **±** **3.3**	**NS**
**BMI (kg/m** ^2^ **)**	**24.** **5±4.5**	**24.3** **±4.5**	**NS**
**Duration of infertility (years)**	**3.** **5±** **2.0**	**3.2** **±** **2** **.** **1**	**NS**
**Duration of stimulation (days)** **Mean gonadotrophin dose (IU)**	**10.38** **±** **5** **.** **12** **2135.3** **±** **647.6**	**10.14** **±** **5** **.** **83** **2118.3** **±** **647.1**	**NS** **NS**
**Day 3 FSH (IU/ml)** **Day 3 E**_2_ ** (pg/ml)** **No. of oocytes retrieved** **No. of mature oocytes**	**6.7** **±** **1.8** **5** **3.5±** **2** **4.** **8** **16** **.** **3** **±** **4** **.** **5** **9.27** **±** **4.8**	**6.8** **±** **1.6** **54** **.** **6** **±** **2** **4.** **2** **16** **.** **1** **±4.** **2** **9.26** **±** **5.1**	**NS** **NS** **NS** **NS**
**Total 2PN number**	**6.73** **±** **4.6**	**6.06** **±** **4.29**	**NS**

Unless otherwise indicated, data are presented as mean ± SD.

NS = not significant (p>0.05)

BMI= body mass index

**Table-II:** Sperm parameters after preparation in all patients

***Parameter***	***C*** ***onventional IVF***	***ICSI***	***P***
**C** **oncentration ** **(×10** ^6^ **/ml** **)**	**7.2 ** **±** ** 1.3**	**6.8 ** **±** ** 1.5**	**NS**
**Sperm progressive motility (%)**	**21.2 ** **±** ** 10.1**	**21.8 ** **±** ** 9.8**	**NS**

**Values are mean ± SD**

**NS = not significant (p>0.05)**

**Table-III:** Comparison of the fertilization rate, embryo quality, implantation rate and pregnancy rate from couples with moderate oligoasthenzoospermia between after IVF and after ICSI

	Conventional IVF	ICSI	P
**Fertilization rate (%)**	**70.1**	**71.9**	**NS**
**Cleavage rate (%)**	**97.4**	**94.7**	**NS**
**Good embryo rate (%)**	24.0*	32.9*	**0.01**
**Implantation rate (%)**	**36.8**	**28.4**	**NS**
**Pregnancy rate (%)**	**37.7**	**41.1**	**NS**

*: P<0.05,

NS = not significant (p>0.05**)**

## DISCUSSION

This study confirms that performing conventional IVF in sibling oocytes in the first cycle for couples with moderate oligoasthenozoospermia did not reduce the major indices compared with after ICSI. It is also an important test of sperm fertilizing ability, to be used as a guideline for the future treatment of choice.

Similar study comparing IVF and ICSI in sibling oocytes from couples with mild male infertility has been reported. The fertilization rate was achieved with both after IVF and ICSI depended on the quality of semen. It has been reported that sperm motility and concentration were better predictor of fertilization potentials than sperm morphology. One study on the effect of sperm motility showed that there was a high risk of absence of fertilization with conventional IVF compared with treated with ICSI. In addition, fertilization rate of ICSI was much better when motile sperm can be selected. These results indicate that the sperm motility might affect the fertilization rate both IVF and ICSI, and the choice of excellent sperm could improve the fertilization rate.

With regard to embryo quality, we found significantly higher good quality embryos after ICSI compared with after IVF in couples with moderate oligoasthenozoospermia. In accordance with previous study, the results showed a significantly higher proportion of morphologically superior embryos and no fragmentation for ICSI in sibling oocytes than for sibling oocytes by IVF. It might suggest that exposure of the IVF embryos to large number of spermatozoa, causing suboptimal culture conditions, affects the quality of embryos. However, other study claimed that embryos obtained by IVF were superior to those obtained by ICSI in term of embryo morphology. It might indicate that ICSI could increase the levels of defects which are likely to have an adverse effect on embryo development or the physiological event of normal spermatozoon-oocyte fusion was changed after injection. In our study, the ICSI treatment might lead to damaged blastocyst development were not confirmed.

In the embryo transfer process, the choice of embryo was based on embryo quality regardless of whether they were derived from either IVF or ICSI in the first cycle. The implantation rate and pregnancy rate were significantly higher quality embryos after IVF compared with after ICSI in this study but these differences were not statistically significant. It is not clear whether this difference is due to damage incurred during the injection process or to a negative effect of overriding. The results were consistent with those of one study which showed in a prospective randomized study, in cases of tubal factor infertility with normal semen, and there was no significant difference in implantation and pregnancy rates between IVF and ICSI. No statistically significant relationship between sperm parameters and implantation rate or pregnancy outcome was found after ICSI. Moreover, no significant difference in clinical pregnancy rate between one embryo transfer and two embryo transfers was also observed.

In this study, some inherent limitations should be considered. Firstly, this study was a retrospective analysis, and there may have introduced selection bias. However, in order to reduce the bias among couples, we formulated the strict inclusion and exclusion criteria for patients. Secondly, different technologists might also be noticed. Semen analysis and assisted reproductive technology were performed by different technologists, whom have been professionally trained and have performed these technologies for several years in our reproductive medical center. Thirdly, the sample sizes are relatively small. Although this study provided statistically significant, further study with large samples are needed to ensure the reliability of the outcomes. Therefore, these potential sources of bias have been avoided as soon as possible in order to guarantee the accuracy of the clinical outcomes, allowing clinical application of the results practically.

In conclusion, this study showed that couples with moderate oligoasthenozoospermia as the only male factor infertility underwent IVF or ICSI, there was no significant difference in the clinical outcomes between conventional IVF and ICSI in the first cycle. As the safety of ICSI is still under evaluation.
